# The effect of frailty on the development of acute kidney injury in critically-ill geriatric patients with COVID-19

**DOI:** 10.55730/1300-0144.5488

**Published:** 2022-07-09

**Authors:** Murat KÜÇÜK, Bişar ERGÜN, Mehmet Nuri YAKAR, Özer Ural ÇAKICI, Erdem YAKA, Bilgin CÖMERT, Ali Necati GÖKMEN, Begüm ERGAN

**Affiliations:** 1 Department of Internal Medicine and Critical Care, Faculty of Medicine, Dokuz Eylül University, İzmir, Turkey; 2 Department of Anesthesiology and Critical Care, Faculty of Medicine, Dokuz Eylül University, İzmir, Turkey; 3 Department of Physiology, Faculty of Medicine, Gazi University,, Ankara, Turkey; 4 Department of Neurology, Faculty of Medicine, Dokuz Eylül University, İzmir, Turkey; 5 Department of Internal Medicine and Critical Care, Medicana International İzmir Hospital, İzmir, Turkey; 6 Department of Pulmonology and Intensive Care, Faculty of Medicine, Dokuz Eylül University, İzmir, Turkey

**Keywords:** Frailty, COVID-19, acute kidney injury, intensive care

## Abstract

**Background/aim:**

Acute kidney injury is strongly associated with mortality in critically ill patients with coronavirus disease 2019 (COVID-19); however, age-related risk factors for acute kidney injury are not clear yet. In this study, it was aimed to evaluate the effects of clinical factors on acute kidney injury development in an elderly COVID-19 patients.

**Materials and methods:**

Critically ill patients (≥65years) with COVID-19 admitted to the intensive care unit were included in the study. Primary outcome of the study was the rate of acute kidney injury, and secondary outcome was to define the effect of frailty and other risk factors on acute kidney injury development and mortality.

**Results:**

A total of 132 patients (median age 76 years, 68.2% male) were assessed. Patients were divided into two groups as follows: acute kidney injury (n = 84) and nonacute kidney injury (n = 48). Frailty incidence (48.8% vs. 8.3%, p < 0.01) was higher in the acute kidney injury group. In multivariate analysis, frailty (OR, 3.32, 95% CI, 1.67–6.56), the use of vasopressors (OR, 3.06 95% CI, 1.16–8.08), and the increase in respiratory support therapy (OR, 2.60, 95% CI, 1.01–6.6) were determined to be independent risk factors for acute kidney injury development. The mortality rate was found to be 97.6% in patients with acute kidney injury.

**Conclusion:**

Frailty is a risk factor for acute kidney injury in geriatric patients with severe COVID-19. The evaluation of geriatric patients based on a frailty scale before intensive care unit admission may improve outcomes.

## 1. Introduction

The World Health Organization (WHO) declared COVID-19 a pandemic on March 11, 2020, after the disease had been detected in 113 countries outside China [1]. The epidemic was literally revolutionary in a regular man’s life, as well as completely subversive to healthcare all around the globe. A new coronavirus was identified as the causative agent of these cases, one that had not been detected previously in humans. The disease was called “Coronavirus Disease 2019 (COVID-19),” and the virus was named “Severe Acute Respiratory Syndrome-Coronavirus 2 (SARS-CoV-2)” due to its close resemblance to a previous SARS coronavirus.

Although all age groups are at risk of being infected with COVID-19, older individuals are at higher risks of serious disease due to aging, immunosenescence, and underlying medical conditions. Increased cardiometabolic diseases, decreased renal function, the use of multiple drugs, physical inadequacies, frailty, neurological disorders, and dementia have made the elderly a particularly affected population during the pandemic [2]. Previous studies have shown that advanced age and the presence of comorbidities are significantly associated with mortality in COVID-19 patients [3,4]. Acute kidney injury (AKI) is a common complication detected in critically ill patients. Kidneys are more susceptible to acute damage, especially at an advanced age [5,6]. Cheng et al. have previously reported that serum creatinine levels at the time of hospital admission are higher in elderly patients (mean age, 73 years) with COVID-19 and that the presence of AKI is associated with poor clinical results [7]. A unique clinical entity, named COVID-19-associated AKI, has also been proposed to be used to name the possible direct effect of the virus on the kidneys [8]. Structural remodeling, vascular sclerosis, increased glomerular sclerosis, and decreased kidney mass, which occur due to aging, are the risk factors for acute kidney injury, in addition to coexisting comorbidities [9]. Although the development of AKI due to viral invasion is still not clearly understood, SARS-CoV-2 nucleocapsid protein antigen has been detected in kidney autopsy samples of COVID-19 patients with renal dysfunction and viruses have been detected in the parenchymal tubular epithelium and podocytes, which is now accepted as direct evidence for viral infection [10,11]. Overall, it is well-accepted that COVID-19 patients are more prone to develop AKI during their clinical course [12].

Frailty is functional impairment caused by cumulative declines across multiple organ systems. It defines an obvious state of vulnerability, and fulfillment of three out of five standardized clinical findings is suggested for the definition of frailty. The criteria comprised loss of grip strength, low energy in daily activities, slowing in waking speed, decreased physical activity, and unintentional weight loss [13,14]. Moreover, validated scales have come into practice to be used in the evaluation of the patients and foresee their outcomes [15]. Previous studies have shown that frailty is an independent risk factor for intensive care unit (ICU) mortality, length of stay, and rehospitalization in critical patients [16,17]. Kader et al. have emphasized the fact that frailty is a risk factor for AKI development in critically ill patients [18]. However, this relationship has not been studied in severe COVID-19 patients. The aim of the present study was to assess the rate of AKI and the independent effect of frailty on AKI development in critically ill geriatric patients with COVID-19.

## 2. Materials and methods

### 2.1. Study population

Consecutive patients aged ≥65 years diagnosed with laboratory-confirmed COVID-19 disease (diagnosis was confirmed with a positive test for reverse transcription-polymerase chain reaction [RT-PCR] in any respiratory sample or clinical, radiological, and laboratory data) and admitted to the ICU between March 2020 and January 2021 were included in the study. Patients <65 years, those who had been previously diagnosed with chronic kidney disease (CKD) Stage 4–5, those who had previously received renal replacement treatment (RRT), and those whose ICU length of stay was <24 h were excluded. This retrospective study was approved by the Local Ethics Committee (with date 29.03.2021 and number 2021/10-37). Informed consent was waived because of the study design.

### 2.2. Data collection

Hospital records and laboratory data for each patient were examined, and the following data were collected: age, sex, comorbidities, onset of symptoms, hospitalization and ICU stay, laboratory parameters on the first day upon admission to the ICU (arterial blood gas [PaO_2_: arterial partial oxygen pressure, PaCO2: arterial partial carbon dioxide pressure, FiO2: fraction of inspired oxygen, PO2/FiO2; HCO3; bicarbonate, SO2; oxygen saturation], complete blood count, C-Reactive Protein [CRP], procalcitonin, lactate dehydrogenase [LDH], alanine aminotransferase [ALT], aspartate aminotransferase [AST], D-dimer, serum creatinine [sCr], total bilirubin, ferritin, and cardiac troponin I), Acute Physiology and Chronic Health Evaluation (APACHE) score II, the Oxygenation Index (OI), Sequential Organ Failure Assessment (SOFA) score, body mass index (BMI), Charlson Comorbidity Index (CCI), complications (septic shock, cardiac injury, new-onset arrhythmia, ventilator-associated pneumonia [VAP], pneumothorax-pneumomediastinum, and acute kidney injury), supportive treatments during hospitalization (mechanical ventilation [MV] data, RRT, and vasoactive drug therapy), length of ICU and hospital stays, and mortality.

### 2.3. Definitions

AKI definition was determined according to the “Kidney Disease: Improving Global Outcomes” (KDIGO) classification system [19]. If sCr levels were within normal limits at the time of admission, this was considered basal sCr; if sCr changed during hospitalization and no sCr was available before admission (within 3 months), the lowest sCr value measured during the hospital stay was considered basal sCr. Considering the retrospective nature of the study and the risk of mistakes and missing data regarding the assessment of urination at frequent intervals included in the KDIGO criteria, only sCr was considered as a criterion for AKI identification. The patients were evaluated in two groups including those that developed AKI (AKI group) and those that did not develop AKI (non-AKI group) during ICU hospitalization.

The use of nephrotoxic drugs at any time prior to AKI development was recorded in the non-AKI and AKI groups. Nephrotoxic drugs were listed such as antihypertensive and nonsteroidal antiinflammatory drugs, contrast substance, aminoglycosides, glycopeptides, and colistin antibiotics, which are thought to affect nephrotoxicity and are frequently used in ICUs.

Sepsis and septic shock were defined according to the 2016 Third International Consensus on the Definition of Sepsis and Septic Shock [20]. In addition, VAP was defined as an increase in the amount of tracheal secretion 48 h after intubation, darkening in color, increased purulence, and ≥10^5^ CFU/mL reproduction in an endotracheal aspiration (ETA) sample [21]. If the troponin I level was above the upper reference limit of the 99^th^ percentile or new abnormalities were shown upon electrocardiography and echocardiography, this patient was considered as having cardiac injury [22]. If the patient experienced respiratory failure or the initial respiratory support therapy failed, this was identified as the escalation of respiratory support treatment. OI is calculated using the formula (FiO_2_ divided by PaO_2_, and the division is multiplied by the mean airway pressure) and used for its role in predicting the clinical outcomes in respiratory failure [23].

The frailty degree was evaluated with the Frail Scale [24]. This scale evaluates fatigue, muscle resistance, aerobic capacity, disease load, and weight loss. Fatigue is mostly evaluated by asking if patients feel tired, and muscle resistance is measured with a report showing the capacity of patients to climb one floor of stairs. The aerobic reserve is identified with independent walking capacity; disease load is defined as having five of a total of eleven diseases (hypertension, diabetes mellitus, cancer [basal-cell carcinoma or equivalent excluded], chronic obstructive pulmonary disease, coronary artery disease or myocardial infarction, congestive heart failure, asthma, arthritis, and cerebrovascular disease chronic kidney disease), and finally, weight loss was defined as having had a decrease of 5% or more in weight in the last 6 months.

Patients were classified as nonfrail for a total score of 0, prefrail for 1–2 points, and frail for ≥3 scores. Frail scale score was calculated using the information from patients or patients’ relatives.

### 2.4. Statistical analysis

All continuous variables were presented as mean ± standard deviation [SDs] or median (inter-quartile ranges [IQRs]), and categorical variables were presented as numbers and percentages (%). For all variables, descriptive statistics were calculated with Student’s t-test, Mann-Whitney U-Test, χ2, or Fisher’s Exact Test. Multivariate logistic regression analysis was performed, using the Enter Method, to investigate the risk factors in AKI development. A p-value of <0.05 was considered statistically significant. The Statistical Package for the Social Sciences (SPSS) Version 26.0 (IBM) was used for all analyses.

## 3. Results

### 3.1. Patient characteristics

A total of 306 critically ill patients who had COVID-19 were admitted to the ICU. Of these, 175 were over the age of 65. Forty-three patients were excluded due to the exclusion criteria, and 132 patients were included in the study (Figure). Acute kidney injury was detected in 84 (63.6%) of them.

The median age of the AKI group was similar to that of the non-AKI Group (76 [70–83] vs. 72 [69–80], p = 0.09). APACHE II score was higher in the AKI group than in the non-AKI group (23 [15–28] vs. 19 [11–23], respectively, p = 0.02). Similarly, the SOFA score, in terms of intensive care admission, was higher in the AKI group than in the non-AKI group (6 [5–7], vs. 4 [3–5], p = 0.02). The number of frail patients was significantly higher in the AKI group than in the non-AKI group (48.8% vs. 8.3%, p ≤ 0.01). Conversely, nonfrail patients were significantly higher in the non-AKI group than in the AKI group (43.8% vs. 19%, p =< 0.01. All clinical data of the cohort is given in Table 1.

### 3.2. Laboratory findings and AKI classification

LDH (596 [492–768] U/L vs. 534 [370–669] U/L, p = 0.02) and D-dimer (1.95 [1.30–4.5] mg/mL vs. 1.35 [0.8–3.2] mg/ml, p = 0.03) were higher in the AKI group than in the non-AKI group.

Among the AKI developing patients, eleven (13.1%) had Stage 1, 16 (19%) had Stage 2 and 57 (67.9%) had Stage 3 AKI. Laboratory data of the cohort is given in Table 2.

### 3.3. Characteristics for ICU stay

The incidence of VAP (63.1%, vs. 31.3%, p < 0.01) and septic shock (91.7% vs. 41.7, p < 0.01) were higher in the AKI group than in the non-AKI group during ICU stay. The increase in respiratory support treatment during ICU hospitalization (59.5% vs. 33.3%, p < 0.01) and the development of vasopressor requirements (83.3% vs. 45.8%, p < 0.01) were significantly higher in the AKI group than in the non-AKI group.

In all patients, ICU mortality was 75% (99/132), and hospital mortality was 76.5% (101/132). In the AKI group, ICU and hospital mortality were significantly higher (97.6% vs. 35.4%, p < 0.01; 97.6% vs. 39.6%, p < 0.01, respectively) than in the non-AKI group. It was also found that 38.2% of the patients in the group that developed AKI received RRT and all died. Additionally, ICU survival was detected in two patients who developed Stage 1 AKI. Among all patients, ICU and hospital mortality was 97.7% (44/45) in frail patients and 48.6% (18/37) in nonfrail patients. In prefrail patients, ICU mortality was 74% (37/50), and hospital mortality was 78% (39/50).

Clinical data involving the course during the ICU stay is given in Table 3.

### 3.4. Multivariate model for AKI development

The variables included in the regression model were APACHE II score, CCI, frailty score, vasopressor requirement, the increase in respiratory support, and nephrotoxic drug use. Frail patients (OR 3.32; 95% CI, 1.67–6.56, p < 0.001), vasopressor requirement (OR 3.06; 95% CI, 1.16–8.08, p = 0.02), and the increase in respiratory support (OR 2.60; 95% CI, 1.01–6.6, p = 0.04) were identified as independent risk factors for AKI development in elderly patients with COVID-19 disease. The results of the multivariate analysis are given in Table 4.

## 4. Discussion

We evaluated the factors that affected AKI development and ICU mortality in elderly patients with COVID-19, and three important results were found. First, the AKI rate was 63.6% in geriatric patients with severe COVID-19 disease. Second, the mortality rate was significantly higher in patients with AKI, while AKI is a relatively common problem in patients >65 years of age with severe COVID-19. Third, frailty alone increased the risk of AKI development three-fold.

Although the incidence of AKI in COVID-19 disease varies between 37% and 46%, it has been reported to increase up to 68% in patients admitted to the ICU [25,26]. Current evidence suggests that the COVID-19 course is complicated by a high risk of AKI [27]. The incidence of AKI in critically ill patients with COVID-19 disease over 65 years of age was 63.6% in our study, and it was found that AKI development was associated with high mortality in the geriatric patient group (97.6%). However, mortality rates have been found differently in patients with AKI in a study conducted by Alessandri et al. in that AKI has been found to have developed in 57% of critical patients with COVID-19 disease, and age has been identified as a significant risk factor for AKI development. In the same study, the mortality rate has been found as 69% in the entire patient group, while it has been determined as 90% in patients with Stage-3 AKI [28]. In another study conducted by Piñeiro et al., the rate of AKI development in the ICU has been found as 22% and the mortality rate was 52% in the patient group with a median age of 72 years [29].

The presence of frailty upon ICU hospitalization in critically elderly patients with COVID-19 disease was identified as an independent risk factor for AKI development in the present study. Frail patients were found to have 100% mortality in the AKI-developing group. Previous studies have reported that frailty is independently associated with ICU mortality (16). In a study in which ICU mortality is 69%, it has been shown that there is a correlation between SOFA and APACHE II scores and frailty, and the survival rates of frail patients are lower at 1 and 6 months after ICU discharge [17].

The scoring systems typically used to determine the prognosis of patients in ICUs are APACHE II and SOFA [30]. However, no specific tests are available for critical elderly patients with which clinical outcomes are predicted. It must be kept in mind that frailty can be an important factor in the evaluation of these patients, especially considering that mortality increases with frailty at significant levels in this age group. We believe that it would be very valuable to assess patients for frailty, in addition to the scores we are currently using. Moreover, it would also be helpful for the decision-making process regarding life-supporting aggressive treatments or palliative care for these patients. While examining the frailty status, we used the Frail Scale considering its evidence-supported role to indicate mortality [31].

Studies examining the effects on AKI development, risk factors and mortality in elderly critical COVID-19 patients are inadequate in the present time. In the study conducted by Li et al., AKI has developed in 59% of elderly critical COVID-19 patients, and mortality has been found as 97.2% [32]. In the same study, invasive mechanical ventilation (IMV) and vasopressor need, and septic shock have been found higher in the group that developed AKI. Furthermore, LDH and D-dimer levels and basal sCr on hospitalization are higher in the group that developed AKI. Similarly, the need for vasopressors was an independent risk factor for AKI development (OR 3.06) in this study. In addition, elevated LDH and D-dimer levels, which are the biomarkers associated with disease severity, were higher in the group that developed AKI at statistically significant levels. In our study, although no significant differences were detected in blood gas values in the ICU hospitalization of patients, OI was lower in the AKI group; however, no significant differences were detected between initial respiratory support treatments in both groups. However, it was determined in the follow-ups that the increase in respiratory support was significantly higher in the AKI group and was an independent risk factor for AKI. When considered from this point of view, it may be that permissive hypoxia has increased effects on sensitive kidneys, and late intubation may increase the risk of AKI development. Although basal and ICU-hospitalization SCr values were higher in the AKI group, no statistically significant differences were detected in our study. However, given that patients with Stage 1–3 chronic kidney disease were more common in the AKI group (16.7% vs. 10.4%), this group may be at a higher risk of AKI development. The incidence of VAP and septic shock was significantly higher in the AKI group. Although the difference between the groups was not statistically significant, the high number of MV days and immunosuppressive treatments in the AKI group may have caused increased VAP incidence.

Our study has certain limitations. First, the study was conducted in the ICU of a single medical center; therefore, the results cannot be generalized. Another limitation was information bias due to the retrospective nature of the study. The frailty status of some patients who could not communicate was assessed according to the data provided by relatives. The present study also has certain strengths. Although AKI and mortality data for severe COVID-19 are present in the literature, specific data for the geriatric population remain limited. Moreover, to the best of our knowledge, this is the first study to assess a new risk factor for AKI development, the frailty score, in geriatric patients with severe COVID-19.

In conclusion, this study examined frailty and other risk factors that affect AKI development in elderly COVID-19 patients admitted to the ICU due to critical disease and found that frailty was an independent risk factor for AKI development. Frailty scale scores must be examined in all elderly patients, particularly in those admitted to ICUs with COVID-19, and further studies must be conducted regarding the use of frailty as a prognostic marker.

## Figures and Tables

**Figure:**
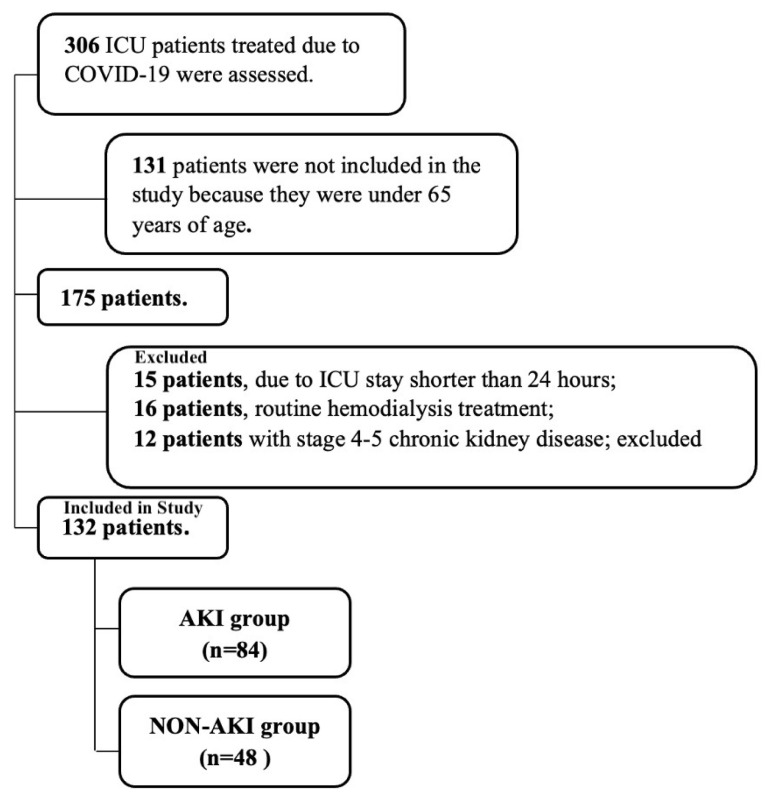
Flowchart of the study.

**Table 1:** Clinical characteristics and comorbidities of older patients with COVID-19.

Characteristic	All Patients (n = 132)	Non-AKI Patients (n = 48)	AKI Patients (n = 84)	p- value
Age (range)	76 (76–83)	72 (69–80)	77 (70–83)	0.09
Sex				
Male (%)	90 (68.2)	28 (58.3)	62 (73.8)	0.06
Female (%)	42 (31.8)	20 (41.7)	22 (26.2)	
Time from symptom onset to ICU admission, days (range)	7 (3–10)	7 (4–9)	7 (3–10)	0.09
Length of hospital stay before ICU admission, days (range)	2 (1–6)	2 (1–6)	2 (1–5)	0.46
SOFA Score^1^ (range)	5 (4–7)	4 (3–5)	6 (5–7)	**0.02**
BMI kg/m^2^ (range)	26 (22–28)	26 (22–27)	26 (22–29)	0.56
CCI (range)	5 (4–7)	5 (3–7)	6 (4–7)	0.06
Frailty Scale				
Nonfrail (%)	37 (28)	21 (43.8)	16 (19)	**<0.01**
Prefrail (%)	50 (37.9)	23 (47.9)	27 (32.1)	0.09
Frail (%)	45 (34.1)	4 (8.3)	41 (48.8)	**<0.01**
Chronic medical illness				
Hypertension (%)	109 (82.6)	39 (81.2)	70 (83.3)	0.81
Diabetes mellitus (%)	48 (36.4)	17 (35.4)	31 (36.9)	1.00
Coronary artery disease (%)	39 (29.5)	11 (22.9)	28 (33.3)	0.23
Congestive heart failure (%)	22 (16.7)	6 (12.5)	16 (19)	0.46
Chronic kidney disease (Stage 1–3) (%)	19 (14.4)	5 (10.4)	14 (16.7)	0.44
Dementia (%)	19 (14.4)	6 (12.5)	13 (15.5)	0.79
Obstructive pulmonary disease (%)	17 (12.9)	7 (14.5)	10 (11.9)	0.06
Malignancy^2^ (%)	14 (10.6)	3 (6.3)	11 (13.1)	0.25
Cerebrovascular disease (%)	11 (8.3)	1 (2.1)	10 (11.9)	0.05
Chronic atrial fibrillation (%)	10 (7.6)	4 (8.3)	6 (7.1)	1.00
Hyperlipidemia (%)	9 (6.8)	2 (4.2)	7 (8.3)	0.48
Parkinson’s disease (%)	6 (4.5)	2 (4)	4 (4)	0.66

All values are expressed as numbers (percentages) or median (interquartile range).

**Abbreviations:** AKI: Acute kidney injury; APACHE: Acute physiology and chronic health evaluation; BMI: Body mass index. CCI: Charlson Comorbidity Index. SOFA: Sequential Organ Failure Assessment score

1 On the day of intensive care unit admission

2 Includes hematological and solid organ malignancies

**Table 2:** Laboratory findings and KDIGO AKI staging of older patients with COVID-19.

Variables	All Patients (n = 132)	Non-AKI Patients (n = 48)	AKI Patients (n = 84)	p- value
Leukocyte 10^3^/UL (range)	11.4 (8–14.8)	10.6 (6.4–9.7)	11.4 (8.2–14.6)	0.74
Lymphocytes 10^3^/UL (range)	0.5 (0.4–0.9)	0.5 (0.4–0.8)	0.5 (0.3–1)	0.94
Hemoglobin gr/dL (range)	12.4 (11.1–13.6)	12.05 (11–13.4)	12.75 (11.1–13.8)	0.21
CRP mg/L (range)	161 (92.2–228)	141 (88–210)	174 (110–241)	0.20
Procalcitonin ng/mL (range)	0.33 (0.15–1.06)	0.24 (0.08–1.07)	0.36 (0.18–1.06)	0.09
LDH U/L (range)	564 (357–827)	534 (370–669)	596 (492–768)	0.02
Ferritin ng/mL (range)	523 (375–827)	481 (210–824)	539 (294–846)	0.12
Troponin ng/mL (range)	30 (11–205)	24 (8–93)	36 (15.6–261)	0.38
ALT U/L (range)	32 (19–54)	32 (22–65)	33 (19–47)	0.40
AST U/L (range)	51 (35–78)	45 (32–88)	51 (37.5–75)	0.60
Total bilirubin mg/dL (range)	0.92 (0.63–1.14)	0.75 (0.60–1.12)	0.95 (0.65–1.18)	0.17
D-dimer μg/mL (range)	1.70 (1.1–3.97)	1.35 (0.8–3.2)	1.95 (1.30–4.5)	0.03
Baseline sCr mg/dL (range)	0.89 (0.7–1.1)	0.87 (0.69–0.92)	0.92 (0.76–1.10)	0.09
sCr on ICU admission mg/dL (range)	1.02 (0.85–1.39)	1.01(0.80–1.19)	1.10 (0.89–1.31)	0.11
PaO_2_/FiO_2_ ratio (range)	117 (96–154)	127 (101–163)	108 (93–151)	0.13
KDIGO AKI staging				
Grade 1 (%)	11 (8.3)	-	11 (13.1)	NA
Grade 2 (%)	16 (12.1)	-	16 (19)	
Grade 3 (%)	57 (43.2)	-	57 (67.9)	
Arterial blood gas analysis				
pH (range)	7.41 (7.32–7.47)	7.43 (7.33–7.47)	7.39 (7.29–7.4)	0.38
PaO_2_ mmHg (range)	65 (55–81)	69 (57–84)	61 (52–76)	0.07
PaCO_2_ mmHg (range)	35 (31–44)	35 (31–45)	35 (30–44)	0.45
FiO_2_ (range)	0.6 (0.5–0.6)	0.6 (0.5–0.6)	0.6 (0.5–0.6)	0.91
SaO_2_ % (range)	92 (88–95)	93 (90–96)	91 (86–94)	0.06
HCO_3_ mmol/L (range)	23 (20–25)	24 (20–26)	23 (20–25)	0.07
Lactate mmol/L (range)	1.95 (1.4–2.95)	1.85 (1.2–3.2)	2.05 (1.42–2.8)	0.35

AKI: Acute kidney injury; ALT: Alanine aminotransferase; AST: Aspartate aminotransferase; PaO_2_: Arterial partial oxygen pressure; PaCO_2_: Arterial partial carbon dioxide pressure; CRP: C-Reactive protein; FiO_2_: Fraction of inspired oxygen; HCO_3_: Bicarbonate; KDIGO: Kidney disease: improving global outcomes; LDH: Lactate dehydrogenase; SO_2_: Oxygen saturation; sCr: Serum creatinine

**Table 3:** Complications, treatments, and outcomes of older patients with COVID-19.

Variables	All Patients (n = 132)	Non-AKI Patients (n = 48)	AKI Patients (n = 84)	p- value
**Complications**				
Septic Shock (%)	97 (73.5)	20 (41.7)	77 (91.7)	<0.01
VAP (%)	68 (51.5)	15 (31.3)	53 (63.1)	<0.01
Arrhythmia (%)	12 (9)	4 (8.3)	8 (9.5)	0.57
Cardiac injury (%)	36 (27.3)	10 (20.8)	26 (31)	0.23
Pneumothorax/pneumomediastinum (%)	19 (14.4)	7 (14.5)	12 (14.2)	0.12
Treatments				
Initial respiratory support treatment				
IMV (%)	51 (38.6)	17 (35.4)	34 (40.5)	0.58
NIMV (%)	9 (6.9)	3 (6.3)	6 (7.1)	1.00
HFNO (%)	39 (29.5)	16 (33.3)	23 (27.4)	0.55
COT (%)	33 (25)	12 (25)	21 (25)	1.00
Escalation in respiratory support Treatment * (%)	66 (50)	16 (33.3)	50 (59.5)	<0.01
RRT (%)	38 (28.8)	0 (0.0)	38 (45.2)	-
Vasopressors^1^ (%)	92 (69.6)	22 (45.8)	70 (83.3)	<0.01
Antivirals (%)	129 (97.7)	48 (100)	81 (96.4)	0.55
IV Corticosteroid^2^ (%)	104 (78.7)	40 (83)	64 (76.2)	0.38
Pulse corticosteroid^3^ (%)	54 (40)	19 (39)	35 (41)	0.85
LMWH (%)	129 (97.7)	46 (95.8)	83 (98.8)	0.29
Nephrotoxic agent use** (%)	38 (28.7)	9 (18.7)	29 (34.5)	0.07
Outcomes				
Duration of IMV, days (range)	9 (4–14)	8 (4–15)	9 (4–14)	0.84
Length of hospital stay, days (range)	16 (11–21)	16(14–21)	15 (10–20)	0.08
Length of ICU stay days (range)	10 (6–16)	8 (5–17)	11 (6–16)	0.18
Hospital mortality (%)	101 (76.5)	19 (39.6)	82 (97.6)	<0.01
ICU mortality (%)	99 (75)	17 (35.4)	82(97.6)	<0.01

All values are expressed as numbers (percentages) or median (interquartile range).

Notes:

1 Norepinephrine > 0.15 Mg/Kg/min

2 Iv Corticosteroid*: intravenous 0.5–1 mg/kg/day

3 Pulse Corticosteroid*:> 250 mg/day

* If the patient is altered in respiratory failure or has failed from HFNO

** Drugs with nephrotoxicity: mainly included aminoglycosides, glycopeptides, and colistin

**Abbreviations:** COT: Conventional oxygen therapy; HFNOT: High flow nasal oxygen therapy; ICU: Intensive care unit; IMV: Invasive mechanical ventilation; IV: Intravenous; NIMV: Noninvasive mechanical ventilation; RRT: Renal replacement therapy; VAP: Ventilator-associated pneumonia

**Table 4:** Risk factors associated with acute kidney injury in the multivariate regression analysis among patients with COVID-19. (95% CI: 95% confident interval).

	OR (95% CI)	p-value
Frailty*	3.32 (1.67–6.56)	<0.001
Use of vasopressors	3.06 (1.16–8.08)	0.02
Escalation in respiratory support	2.60 (1.01–6.6)	0.04
Charlson comorbidity index	1.00 (0.81–1.23)	0.98
APACHE II score	1.02 (0.96–1.09)	0.36
Nephrotoxic agent use	1.5 (0.56–4.03)	0.41

**Abbreviations:** APACHE; Acute Physiology and Chronic Health Evaluation

* Multivariate analysis carried out grouping the fraile vs. not fraile (including prefrail and nonfrail) patients of the cohort.
